# Risk factors of gastrointestinal bleeding after cardiopulmonary bypass in children: a retrospective study

**DOI:** 10.3389/fcvm.2023.1224872

**Published:** 2023-09-19

**Authors:** Zheng-Qing Li, Wei Zhang, Zheng Guo, Xin-Wei Du, Wei Wang

**Affiliations:** Department of Cardiothoracic Surgery, Shanghai Children’s Medical Center, School of Medicine, Shanghai Jiao Tong University, Shanghai, China

**Keywords:** congenital heart disease, cardiopulmonary bypass, children, gastrointestinal bleeding, risk factors

## Abstract

**Background:**

During cardiac surgery that involved cardiopulmonary bypass (CPB) procedure, gastrointestinal (GI) system was known to be vulnerable to complications such as GI bleeding. Our study aimed to determine the incidence and risk factors associated with GI bleeding in children who received CPB as part of cardiac surgery.

**Methods:**

This retrospective study enrolled patients aged <18 years who underwent cardiac surgery with CPB from 2013 to 2019 at Shanghai Children's Medical Center. The primary outcome was the incidence of postoperative GI bleeding in children, and the associated risk factors with postoperative GI bleeding episodes were evaluated.

**Results:**

A total of 21,893 children who underwent cardiac surgery with CPB from 2013 to 2019 were included in this study. For age distribution, 636 (2.9%) were neonates, 10,984 (50.2%) were infants, and 10,273 (46.9%) were children. Among the 410 (1.9%) patients with GI bleeding, 345 (84.2%) survived to hospital discharge. Incidence of GI bleeding in neonates, infants and children were 22.6% (144/636), 2.0% (217/10,984) and 0.5% (49/10,273), respectively. The neonates (22.6%) group was associated with highest risk of GI bleeding. Patients with GI bleeding showed longer length of hospital stays (25.8 ± 15.9 vs. 12.5 ± 8.9, *P* < 0.001) and higher mortality (15.9% vs. 1.8%, *P* < 0.001). Multivariate logistic regression analysis showed that age, weight, complicated surgery, operation time, use of extracorporeal membrane oxygenation (ECMO), low cardiac output syndrome (LCOS), hepatic injury, artery lactate level, and postoperative platelet counts were significantly associated with increased risk of GI bleeding in children with congenital heart disease (CHD) pediatric patients that underwent CPB procedure during cardiac surgery.

**Conclusion:**

The study results suggest that young age, low weight, long operation time, complicated surgery, use of ECMO, LCOS, hepatic injury, high arterial lactate level, and low postoperative platelet counts are independently associated with GI bleeding after CPB in children.

## 1. Introduction

In data from previous studies, 6–9 of every 1,000 newborns were diagnosed with different forms of congenital heart disease (CHD), in which majority require surgical correction during childhood (1, 2). The gastrointestinal (GI) system is most vulnerable to complications (i.e., GI bleeding) after cardiac surgery due to low abdominal perfusion after prolonged ischemia (3, 4). Despite advanced improvements in CPB techniques, anesthesia procedure, and intensive care support, GI bleeding remains a serious complication after cardiopulmonary bypass (CPB), with incident rate ranging from 0.2% to 2%, with a significantly high mortality rate observed between 8.8% and 19.0% (3–13). Despite the apparent association in between postoperative GI bleeding with length of intensive care unit stays and increased morbidity, risk factors associated with GI bleeding after CPB in children remains unclear (14–17).

Our study aimed to determine risk factors of GI bleeding after CPB in children aged <18 years. We extensively collected potential preexisted factors and variables during whole course of hospitalization. By identifying potential risk factors, this might help surgeons to deploy preventive measures for high-risk patients and enhance early recognition of postoperative GI bleeding, thus improving prognostic outcomes in this cardiovascular surgical population.

## 2. Methods

### 2.1. Patient and study design

This retrospective study was carried out and approved by the Ethical Committee of Shanghai Children's Medical Center. Medical files of all patients who underwent cardiac surgery with CPB from January 2013 to December 2019 were collected from database and analyzed. Inclusion criteria refers to patients <18 years of age who underwent cardiac surgery with CPB. Condition such as cardiac surgery without CPB procedures, incomplete medical records or GI bleeding occurs within 30 days prior to cardiac surgery were excluded. Variables investigated in the study include the preexisted demographic data, laboratory test data, and surgical related variables. All these study data were obtained from electronic medical record system of Shanghai Children's Medical Center. This study was approved by the Ethical Committee of Shanghai Children's Medical Center.

### 2.2. Definition and variables

We retrospectively reviewed 21,893 consecutive cardiac surgery patients. Cardiac surgical procedure refers to any cardiac or intrathoracic great vessel procedure. GI bleeding refers to hemorrhage from the upper or lower GI tract. The clinical presentation of GI bleeding varies according to severity and localization of bleeding. Definition of GI bleeding refers to a positive occult blood test (OB) results in specimen including feces or gastric juice.

The following data were recorded for each cardiac surgical procedure: patient demographics [age, gender, premature birth (birth <37 weeks)], operative procedure, CPB time (minutes), temperature of CPB, perfusion mode of CPB, and postoperative factors [(length of hospital stay, length of mechanical ventilation, use of renal replacement therapy, and use of ECMO, postoperative ejection fraction, postoperative dialysis, postoperative lactate, postoperative hepatic function (ALT and AST), postoperative platelet, and survival to hospital discharge (%)]. Patients' age was classified into three categories: neonates (<28 days), infants (29 days to 1 year), and children (1–18 years). Operative procedure risk stratification was categorized using the Risk Adjusted Classification for Congenital Heart Surgery (RACHS-1), and RACHS-1 scoring 3–6 was defined as complicated surgery. In our study, hepatic injury was defined as detection of ALT ≥ 80 U/L and/or AST ≥ 80 U/L in 30 days after surgery. Low cardiac output syndrome (LCOS) refers to a decrease in cardiac output that is due to myocardial dysfunction. Diagnostic criteria for LCOS: Cardiac output index (CI) <2.5 L·min^−1^·m^−2^.

### 2.3. Statistical analysis

All statistical analyses were performed using SPSS 22.0. The measurement data were expressed by mean ± standard deviation $\left(\bar{x}\pm s\right)$, the mean between the two groups was compared by Mann-Whitney *U*-test, the count data were expressed by the number of cases (constituent ratio), and the comparison between groups was expressed by the *χ*^2^ test. Logistic regression analysis was applied to assess the association between potential risk factors and GI bleeding after cardiopulmonary bypass. A *P* value of 0.05 was considered statistical significant. Based on the risk factors obtained from the multivariate logistic analysis, the Receiver Operating Characteristic Curve (ROC) were plotted for each risk factor to predict GI bleeding after CPB in Children. Risk prediction models were developed, column line plots were generated using R software package. The discriminative ability of nomograms was evaluated by ROC curve and Area under the Curve (AUC).

## 3. Results

### 3.1. Patient characteristics

A total of 21,893 children who underwent cardiac surgery with cardiac pulmonary bypass from January 1 2013 to December 31 2019 were included in this study. At the time of operation, 636 (2.9%) were neonates, 10,984 (50.2%) were infants, and 10,273 (46.9%) were children. Of 21,893 children, 410 (1.9%) developed GI bleeding as a post-operative complication. Regarding outcomes of patients with GI bleeding, 65 patients (15.9%) died during their hospital stay, while 345 patients (84.1%) survived to hospital discharge. The incidence of GI bleeding in neonates, infants and children were 22.6% (144/636), 2.0% (217/10,984) and 0.5% (49/10,273), respectively. As shown in Table 1, in the non-GI bleeding group, the top 5 congenital heart diseases were ventricular septal defect (44%), atrial septal defect (10.5%), tetralogy of Fallot (8.3%), double outlet of right ventricle (3.6%) and atrioventricular septal defect (3.3%). While in the GI bleeding group, the top 5 congenital heart diseases were coarctation of aorta (25.4%), transposition of great arteries (14.9%), ventricular septal defect (10.0%), anomalous pulmonary venous connection (8.3%) and pulmonary atresia (6.1%). There are statistical differences in length of hospital stay and discharge status between different age groups with GI bleeding or no GI bleeding (Table 2). Patients with GI bleeding after CPB was associated with higher mortality (15.9% vs. 1.8%) compared to non-GI bleeding patients. The overall 30-day mortality rate was 2.0% (443/21,893).

**Table 1:** Preoperative diagnosis of congenital heart diseases in patients with GI bleeding or without GI bleeding.

	GI bleeding (*n *= 410)	NO GI bleeding (*n *= 21,483)
VSD	41 (10.0%)	9,453 (44.0%)
ASD	0 (0.0%)	2,246 (10.5%)
TOF	19 (4.6%)	1,790 (8.3%)
DORV	22 (5.4%)	763 (3.6%)
AVSD	11 (2.7%)	713 (3.3%)
APVC	34 (8.3%)	667 (3.1%)
CoA	104 (25.4%)	575 (2.7%)
TGA	61 (14.9%)	577 (2.7%)
PA	25 (6.1%)	610 (2.8%)
PS	14 (3.4%)	433 (2%)
TVD	2 (0.5%)	446 (2.1%)
MVD	11 (2.7%)	432 (2.0%)
SV	13 (3.2%)	332 (1.5%)
CAD	13 (3.2%)	188 (0.9%)
SRVOT	1 (0.2%)	157 (0.7%)
SLVOT	2 (0.5%)	124 (0.6%)
PTA	11 (2.7%)	75 (0.3%)
CT	2 (0.5%)	69 (0.3%)
PVS	7 (1.7%)	52 (0.2%)
PAS	1 (0.2%)	57 (0.3%)
HLHS	2 (0.5%)	4 (0.0%)
Others	14 (3.4%)	1,720 (8%)

Abbreviations related to the diagnosis of congenital heart diseases are shown in Supplementary Table S1.

**Table 2:** Hospital stay and discharge status comparison between GI bleeding or without GI bleeding in different age group.

	GI bleeding	NO GI bleeding	*P* value
Neonate			
Number	144 (22.6%)	492 (77.4%)	
Hospital stay (days)	26.0 ± 16.1	18.5 ± 10.2	<0.001
Discharge status			0.165
Death	14 (9.7%)	71 (14.4%)	
Live	130 (90.3%)	421 (85.6%)	
Infant			
Number	217 (2.0%)	10,767 (98.0%)	
Hospital stay (days)	24.2 ± 15.1	13.3 ± 8.3	<0.001
Discharge status			<0.001
Death	42 (19.4%)	193 (1.8%)	
Live	175 (80.6%)	10,574 (98.2%)	
Child/adolescent			
Number	49 (0.5%)	10,224 (99.5%)	
Hospital stay (days)	32.0 ± 17.7	11.4 ± 9.3	<0.001
Discharge status			<0.001
Death	9 (18.4%)	113 (1.1%)	
Live	40 (81.6%)	10,111 (98.9%)	

### 3.2. Univariate analysis for GI bleeding

To analysis independent variables and features associated with GI bleeding after CPB, all enrolled patients were categorized into GI bleeding group (*n* = 410) and non-GI bleeding group (*n* = 21,483) based on the post-operative occult blood test results. In consideration of pathophysiology of GI bleeding, types of surgical procedures and findings interpreted from preexisted studies, a series of preoperative, intraoperative and postoperative variables were compared between the two groups.

Results of univariate analysis for GI bleeding after CPB were shown in Table 3. In this CPB cohort (21,893 children), 410 (1.9%) had postoperative GI bleeding. In regards of demographic and clinical variables, patients with GI bleeding were of younger age (278.6 ± 712.1 vs. 720.0 ± 931.6 days, *P* < 0.001) and lower weight. Incidence of preterm birth was more prevalent in the GI-bleeding group, nearly five times (5.4% vs. 1.1% *P* < 0.001) higher than non-GI-bleeding group. Pre-operative assessment such as surgical options (radical or palliative) and RACHS-1 category that largely depends on CHD diagnosis, were significantly different between the two groups. During operation, patients with GI bleeding has longer CPB time (131.8 ± 87.2 vs. 68.8 ± 46.4, *P* < 0.001), aortic cross-clamp time (73.9 ± 40.3 vs. 39.5 ± 38.6 *P* < 0.001) and operation time (252.9 ± 110.9 vs. 151.8 ± 65.0 *P* < 0.001).

**Table 3:** Univariate analysis for gastrointestinal bleeding after cardiopulmonary bypass in children.

	GI bleeding (*n* = 410)	NO GI bleeding (*n* = 21,483)	*P* value
Pre-operative variables			
Age (days)	278.6 ± 712.1	720.0 ± 931.6	<0.001
Age group			
Neonate	144 (35.1%)	492 (2.3%)	<0.001
Infant	217 (52.9%)	10,767 (50.1%)	
Child/adolescent	49 (12.0%)	10,224 (47.6%)	
Gender			0.002
Male	258 (62.9%)	11,887 (55.3%)	
Female	152 (37.1%)	9,596 (44.7%)	
Premature birth (<37 weeks)			<0.001
Yes	22 (5.4%)	227 (1.1%)	
No	388 (94.6%)	21,256 (98.9%)	
Weight (kg)	5.1 ± 6.1	9.5 ± 8.1	<0.001
Pre-operative SpO_2_	88.5 ± 11.5	95.3 ± 18.7	<0.001
Intra-operative variables			
Surgical options			<0.001
Radical surgery	331 (80.7%)	19,519 (90.9%)	
Palliative surgery	79 (19.3%)	1,964 (9.1%)	
RACHS-1 category			<0.001
1–2	57 (13.9%)	6,808 (31.7%)	
3–6	353 (86.1%)	14,675 (68.3%)	
Operation time (min)	252.9 ± 110.9	151.8 ± 65.0	<0.001
CPB mode			<0.001
Parallel	24 (5.9%)	1,008 (4.7%)	
Circulatory arrest	39 (9.5%)	193 (0.9%)	
Selective cerebral perfusion	55 (13.4%)	393 (1.8%)	
Full-flow extracorporeal circulation	292 (71.2%)	19,889 (91.1%)	
CPB temperature			<0.001
Normothermic	72 (17.6%)	11,106 (51.7%)	
Mild hypothermia	152 (37.1%)	8,250 (37.8%)	
Moderate hypothermia	137 (33.4%)	1,838 (8.6%)	
Deep hypothermia	49 (12.0%)	289 (1.3%)	
CPB time (min)	131.8 ± 87.2	68.8 ± 46.4	<0.001
Aortic cross-clamp time (min)	73.9 ± 40.3	39.5 ± 38.6	<0.001
Postoperative variables			
Hepatic injury			<0.001
Yes	73 (17.8%)	366 (1.7%)	
No	337 (82.2%)	21,117 (98.3%)	
Post-operative ECMO			<0.001
Yes	42 (10.3%)	36 (0.2%)	
No	368 (89.7%)	21,447 (99.8%)	
LCOS			<0.001
Yes	230 (56.1%)	1,927 (9.0%)	
No	180 (43.9%)	19,556 (91.0%)	
Ventilation time (min)	278.8 ± 233.0	83.3 ± 154.0	<0.001
Postoperative platelet (10^9^/L)	87.5 ± 64.1	187.2 ± 73.2	0.012
Artery lactate level (mmol/L)	2.72 ± 2.4	1.2 ± 2.8	<0.001
ICU stay (days)	11.8 ± 13.3	6.4 ± 63.0	<0.001
Hospital stay (days)	25.8 ± 15.9	12.5 ± 8.9	<0.001
Discharge status			<0.001
Death	65 (15.9%)	378 (1.8%)	
Live	345 (84.1%)	21,105 (98.2%)	

CPB, cardiopulmonary bypass; ECMO, extracorporeal membrane oxygenation; GI, gastrointestinal; ICU, intensive care unit; LCOS, low cardiac output syndrome; RACHS-1, risk adjustment for congenital heart surgery-1.

As to postoperative platelet counts, patients with GI bleeding had lower level of platelet counts (87.5 ± 64.1 vs. 187.2 ± 73.2, *P* = 0.012). Postoperative artery lactate level was higher in patients with GI bleeding compared with those who did not develop GI bleeding (2.72 ± 2.4 vs. 1.2 ± 2.8, *P* < 0.001).

Overall, patients with GI bleeding had longer hospital stays (25.8 ± 15.9 vs. 12.5 ± 8.9, *P* < 0.001) and intensive care unit (ICU) stays (11.8 ± 13.3 vs. 6.4 ± 63.0, *P* < 0.001). Mortality rate was significantly higher among patients with GI bleeding compared with those who did not develop GI bleeding (15.9% vs. 1.8%, *P* < 0.001).

### 3.3. Multivariate logistic regression analysis of risk factors

The results of multivariate analysis of risk factors for GI bleeding after CPB in children were shown in Table 4 Multivariate analysis showed that the operation time [95% confidence interval (CI): 1–1.004; *P* = 0.01], age (95% CI: 0.999–1.000; *P* < 0.001), complicated surgery (95% CI: 2.304–5.211; *P* < 0.001), weight (95% CI: 0.912–0.988; *P* = 0.011), low cardiac output syndrome (LCOS) (95% CI: 2.203–3.945 *P* < 0.001), use of ECMO (95% CI: 2.736–9.508; *P* < 0.001), hepatic injury (95% CI: 1.05–2.257 *P* = 0.027), artery lactate level (95% CI: 1.194–1.426; *P* < 0.001), and postoperative platelet counts (95% CI: 0.989–0.994; *P* < 0.001) were associated with GI bleeding in CHD children with CPB.

**Table 4:** Multivariate logistic regression analysis of risk factors for gastrointestinal bleeding after cardiopulmonary bypass in children.

	*P* value	Odds ratio	95% CI
Operation time (min)	0.01	1.002	1.000–1.004
Age (days)	<0.001	0.999	0.999–1.000
RACHS-1 category	<0.001	3.465	2.304–5.211
Weight (kg)	0.011	0.95	0.912–0.988
LCOS	<0.001	2.948	2.203–3.945
Hepatic injury	0.027	1.54	1.05–2.257
Artery lactate level (mmol/L)	<0.001	1.305	1.194–1.426
Postoperative platelet (10^9^/L)	<0.001	0.992	0.989–0.994
Postoperative ECMO	<0.001	5.1	2.736–9.508

CI, confidence interval; ECMO, extracorporeal membrane oxygenation; LCOS, low cardiac output syndrome; RACHS-1, risk adjustment for congenital heart surgery-1.

### 3.4. ROC curve of independent risk factor predicting GI bleeding

**Figure 1:**
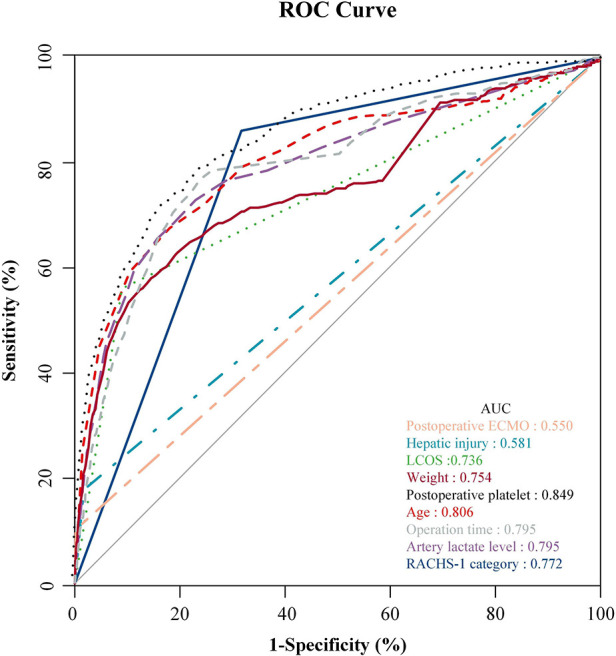
Incidence prediction of GI bleeding based on ROC analysis of age, operation time, RACHS-1 category, weight, LCOS, hepatic injury, artery lactate level, postoperative platelet and postoperative ECMO. The area under the curve of ROC predicting GI bleeding risk after CPB in children.

The independent risk factors obtained from the multivariate logistic analysis are further visually presented through ROC curves (Figure 1). Incidence prediction of gastrointestinal bleeding based on ROC analysis of age, operation time, RACHS-1 category, weight, LCOS, hepatic injury, artery lactate level, postoperative platelet and postoperative ECMO. The area under the curve of ROC predicting GI bleeding risk after cardiopulmonary bypass in children. Table 5 displays the specific details of each ROC curve.

**Table 5:** Details of the ROC curves analysis.

	Area under the curve	Sensitivity (%)	Specificity (%)	Youden index (%)	*P* value	95% CI		Best cut-off value
Postoperative ECMO	0.55	10.2	99.8	10.1	<0.001	0.52	0.581	/
Hepatic injury	0.581	17.8	98.3	16.1	<0.001	0.549	0.612	/
LCOS	0.736	56.1	91.0	47.1	<0.001	0.706	0.765	/
Weight (kg)	0.754	58.0	85.5	43.5	<0.001	0.726	0.782	5.35
RACHS-1 category	0.772	86.1	68.3	54.4	<0.001	0.752	0.792	/
Artery lactate level (mmol/L)	0.795	72.9	77.0	49.9	<0.001	0.769	0.821	1.35
Operation time (min)	0.795	76.8	76.2	53.0	<0.001	0.77	0.82	164
Age (years)	0.808	67.6	82.0	49.5	<0.001	0.782	0.834	0.35
Postoperative platelet (10^9^/L)	0.849	72.0	83.2	55.1	<0.001	0.829	0.868	111.5

CI, confidence interval; ECMO, extracorporeal membrane oxygenation; LCOS, low cardiac output syndrome; RACHS-1, risk adjustment for congenital heart surgery-1.

### 3.5. Nomograms predicting GI bleeding risk after cardiopulmonary bypass in children

**Figure 2:**
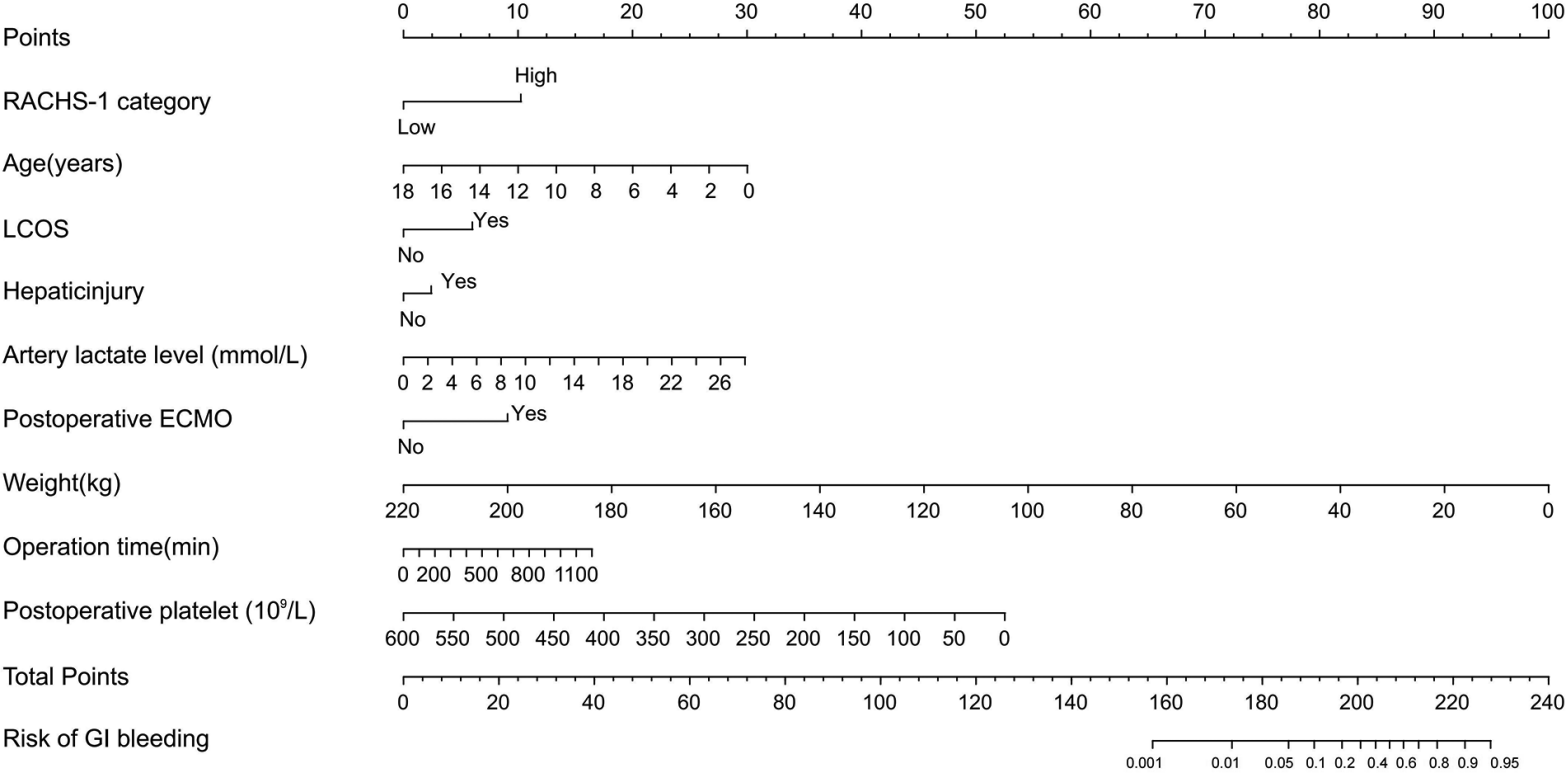
Nomograms predicting GI bleeding risk after cardiopulmonary bypass in children.

**Figure 3:**
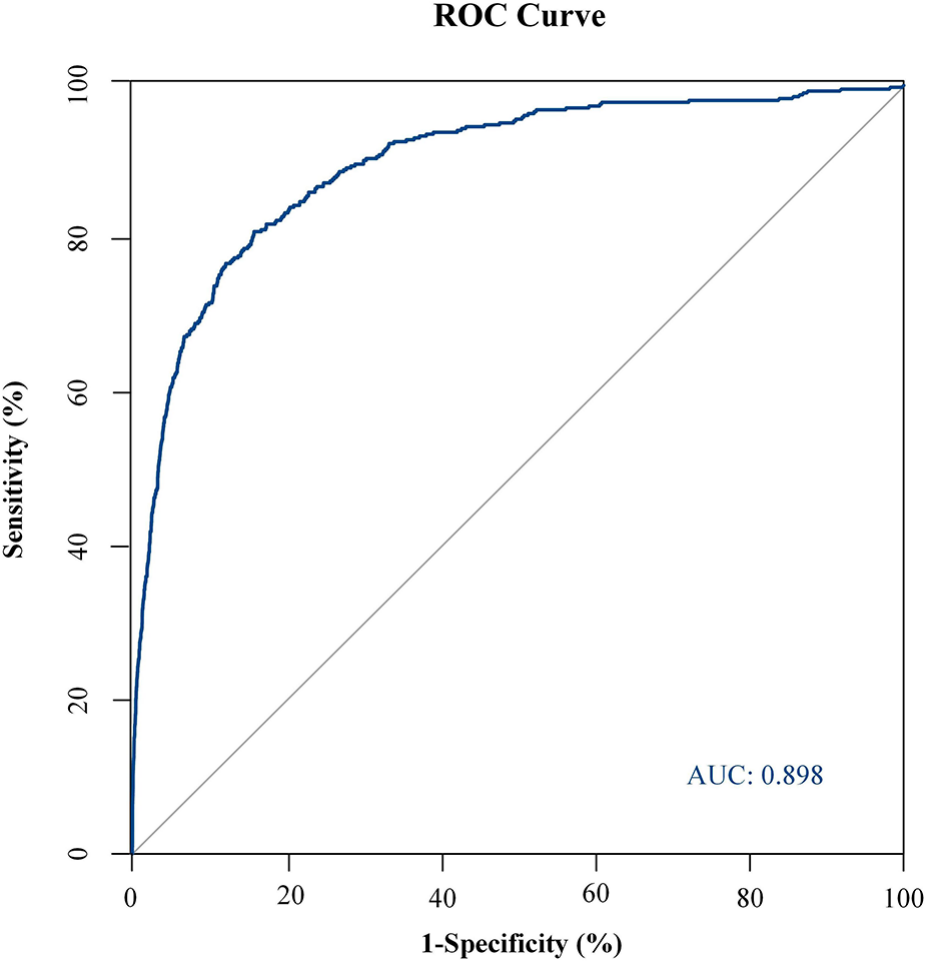
ROC curve of GI bleeding risk prediction model. The area under the ROC curve of the nomogram prediction model for GI bleeding was 0.898 (95% CI: 0.884–0.914, *P* < 0.001), with a sensitivity of 80.9% and specificity of 84.2%.

Probability of GI bleeding after CPB in children can be estimated with the nomograms (Figure 2). In order to predict GI bleeding risk after CPB in children with congenital heart disease, each parameter has a corresponding score on the point axis, and the sum of the scores is plotted on the “total point” axis. The probability of GI bleeding risk after cardiopulmonary bypass in children is the value at a vertical line from corresponding total points. As shown in Figure 3, the area under the ROC curve of the nomograms prediction model for GI bleeding was 0.898 (95% CI: 0.884–0.914, *P* < 0.001), with a sensitivity of 80.9% and specificity of 84.2%.

## 4. Discussion

CHD has gradually turned into the most common congenital defect, in which majority situation require cardiac surgery correction with cardiopulmonary bypass. The ultimate goal of CPB is to maintain sufficient tissue perfusion of all organs and tissues, but it is difficult to fully simulate normal blood circulation and pulsation processes. During this period, due to the redistribution of blood perfusion, blood flow is redistributed and its pathophysiological changes are similar to those observed in shock patients (18–21). Cardiopulmonary bypass give priority to guarantee effective blood supply to the brain and other important organs, which in turn leading to progressive reduction in gastrointestinal mucosa blood flow. GI bleeding after CPB is an unusual fatal event. GI bleeding was found to occur after 0.2%–2% of cardiac surgery cases with CPB and was associated with high mortality rates (8.8%–19.0%) (3–13). In our study, the observed GI bleeding incidence rate was 1.9% (410/21,893), which was similar to that reported in preexisted literature. The mortality rate in this series, 15.9%, well agreed with those previously reported (3–13). We observed a nine-fold increase in mortality risk (15.9% vs. 1.8%, *P* < 0.001) and a significantly longer hospital stay (26.0 ± 15.9 vs. 12.5 ± 8.9, *P* < 0.001) in patients with postoperative GI bleeding. Generally, children undergoing congenital heart surgery were confronted with increased risk of GI complications than adults (22, 23). Frequent monitoring for symptoms and related clinical indicators in these high-risk patients are necessary. Due to the absence of typical clinical signs or masking by signs of other more common complications, GI bleeding demands early clinical recognition so to secure favorable outcomes.

In this large cohort study, we aimed to identify potential risk factor of GI bleeding after CPB by multivariate logistic regression model. Clinical, laboratory and surgical variables during perioperative period were considered. Increased surgical complexity (OR = 3.465, *P* < 0.001) was one of the independent risk factor for GI bleeding. We also observed significant longer CPB time and aortic cross-clamp time in the GI-bleeding group, however multivariate analysis failed to prove its association with GI complication (not shown in table). In earlier studies, CPB time and aortic cross-clamp durations were widely suggested as influential factors of abdominal perfusion. Some evidences pointed out that splanchnic perfusion during CPB procedure might led to inadequate metabolic supply that further enhance GI complications. As reported by Andersson et al. (24), CPB procedure exceeding 150 min was one of the independent factors for predicting GI complications after cardiac surgery. In another prospective study, patients who received CPB longer than 100 min showed significant increase in gut permeability and gut impairment (25). Others conclude the associated risk of cardiopulmonary bypass time on intestinal ischemia damage by measuring biomarkers (26). Though ample evidences mentioned longer CPB time as risk factor of GI complication. However, most of these evidences focus on outcomes such as intestinal ischemic damages but not solely gastrointestinal bleeding. In other words, we cannot fully agree the independent role of extracorporeal circulation variables (such as CPB and aortic cross-clamp duration) in GI bleeding.

In our circulation system, GI system account to 20%–25% of the body's cardiac output and provide 20% of the oxygen supply in adult. Pathogenesis of GI complication could be multifactorial, while major contributing mechanisms during cardiac surgery is reduced systemic blood flow, which leads to insufficient oxygen delivery and energy deficit. Our study findings suggest that a LCOS (95% CI: 2.203–3.945, *P* < 0.001) after surgery is associated with GI bleeding. LCOS was a common postoperative complication, occurring in 9.9% (2,157/21,893) of the patients in this study in the first 24 h after surgery and we observed increased mortality of 6.4% (139/2,157) in this particular subgroup (not shown in table).

In our results, there was a statistical difference in pre-operative SpO_2_ between the bleeding and non-bleeding groups (88.5 ± 11.5 vs. 95.3 ± 18.7, *P* < 0.001), but SpO_2_ was not a risk factor after multivariate analysis. In our analysis of preoperative diagnosis (Table 1), the proportion of TOF in the bleeding group and the non-pulmonary bleeding group was (4.6% vs. 8.3%), respectively. Most congenital heart conditions in cyanotic CHD are complex congenital heart conditions. There are few studies of hypoxia tolerance between cyanotic and acyanotic CHD, and we need to further explore the relationship between them (27, 28). Recently, it has been suggested that cyanotic patients with CHD are characterized by increased arterial stiffness, expressed as a worse vasodilator response (29).

Among all organs, the gut is extremely vulnerable to ischemic injuries during postoperative LCOS because the splanchnic circulation is susceptible to endogenous and exogenous catecholamines. Immobilization, administration of high doses of opioid drugs, and delayed or absent enteral alimentation can aggravate the adverse effects of intraoperative mucosal damage caused by on pump surgery (30, 31). More importantly, prolonged and severe hypoperfusion might result in insufficient splanchnic blood flow. Pathophysiological events include uneven blood flow distribution, oxygen supply abnormalities, oxygen demand imbalance, and systemic inflammation (25, 32). Consequently, impairment of intestinal barrier integrity gradually results in translocation of bacteria and toxins, systemic inflammatory response syndrome and finally remote organ injury. We showed that the GI bleeding following cardiac surgery was common in neonates but rare in children. This may cause by neonates likelier to have LCOS and the sensitivity of neonatal gastrointestinal (33). In our study, we found patients with GI bleeding were younger than non-GI bleeding cases (278.6 ± 712.1 vs. 712.0 ± 931.6). Multivariate logistic regression analysis suggested that a younger age is closely associated with a higher incidence of GI bleeding. Our present series showed an incidence of postoperative GI bleeding of 22.6% (144/636) in neonates, 2.0% (217/10,984) in infants, and 0.5% (49/10,273) in children, which are similar to results reported in earlier literature. As in neonates, low body weight possess increased risk in development of GI bleeding, particularly necrotizing enterocolitis (NEC) (33, 34). Low body weight usually indicates preterm with organ immaturity. Poor visceral regulation to hypoperfusion changes might be the underlined mechanism.

The splanchnic circulation plays an important role during hypovolemia and possess several regulation mechanisms. For instance, splanchnic vasoconstriction stimulated by catecholamine and renin-angiotensin help compensate systemic circulation by increasing total systemic vascular resistance and auto-transfusion (35). Splanchnic hypoperfusion also ensure vital organ perfusion. In this situation, splanchnic flow respond slow to reperfusion even when systemic flow was restored. In more vulnerable patients, persistent hypoperfusion might result to splanchnic ischemia and visceral organ injuries such as GI bleeding. Physically, intestinal villous structure is highly sensitive to ischemia. During perfusion, arterial inflow enters from the base of intestinal villous which result in lower oxygen partial pressure at the tip. However, the high metabolic rate and oxygen demand observed at the tip make it incompatible to ischemia (35, 36).

Another important mechanism is the negative consequences of nonpulsatile blood flow during ischemia (32, 37). Earlier study pointed out that nonpulsatile blood flow as a determinant for renin release, activation of the renin–angiotensin–aldosterone axis and secretion of angiotensin II (38). Hypothermia is also associated with vasoconstriction and altered regional blood flow and distribution. Routine post-operative vasoactive drugs such as noradrenaline and vasopressin are also associated with splanchnic hypoperfusion.

Spotnitz et al. (39) first mentioned the importance of prolonged mechanical ventilation as an indeterminate risk factor for GI complications following cardiac surgery. D'Ancona et al. (7) reemphasized the significance of prolonged postoperative mechanical ventilation on GI bleeding and confirmed as an independent predictor based on multivariate analysis. Other studies have also observed prolonged ventilation as a risk factor during CPB surgery (8, 9, 13, 40, 41). Patients receiving mechanical ventilation often present signs of sympathetic nervous system activation and decreased cardiac output (42), which further contribute to splanchnic hypoperfusion and the injury of gastrointestinal mucosa. Elizalde et al. (43) have clarified the relationship of gastric mucosal ischemia and mechanically ventilated patients in the ICU. Nevertheless, it is reasonable to speculate that mechanical ventilation may enhance the adverse effects of critical illness on GI pathology. Similarly, our study showed a statistical difference in the length of mechanical ventilation between the GI-bleeding group and the non-GI bleeding group (278.8 ± 233.0 vs. 83.3 ± 154.0). However, multivariate analysis verified that long-term mechanical ventilation is not an independent risk factor for GI bleeding (*P* = 0.438).

The application of ECMO has saved the lives of many patients with cardiopulmonary insufficiency, but ECMO-related complications have also become thorny problems in clinical practice. The most common complications are bleeding and thrombus events. To a large extent, the occurrence of such complications is related to anticoagulation management during ECMO. At present, heparin is widely used in clinic, which mediates anticoagulation through its interaction with anti-thrombin (44–46). The results of our study suggest that post-operative use of ECMO (*P* < 0.001) may be a risk factor GI bleeding after CPB in children. This reminds us to pay attention to the effects of heparin during ECMO use and closely monitor relevant indicators such as APTT (activated partial thromboplastin time). At present, there is a shortage of specific anticoagulant preparations for children. Heparin has many disadvantages, such as binding to other plasma proteins and endothelial cells in addition to anti-thrombin, causing unpredictable reactions, difficulties in monitoring, and the risk of heparin-induced thrombocytopenia (HIT). HIT is a life-threatening complication of heparin exposure. HIT is a life-threatening complication of heparin exposure. It is initiated by immunoglobulin G (IgG) against the PF4–heparin complex (47, 48). Currently, a few studies confirm that direct thrombin inhibitors (DTIs), such as bivalirudin, may be a good option for anticoagulation (47–52). High-quality randomized controlled studies are needed to confirm the superiority of bivalirudin to provide a better option for future ECMO anticoagulation.

In order to minimize GI complications, high-risk patients must be identified to ensure optimal splanchnic perfusion. Intraoperative monitoring of gastrointestinal mucosal blood perfusion is essential to avoid gastrointestinal complications. However, splanchnic perfusion is currently difficult to monitor and optimal splanchnic perfusion was not well-defined. Although methods for detecting gastrointestinal mucosal blood flow have been proposed, their effectiveness will be difficult to prove due to the relatively low incidence of gastrointestinal complications. Further research on the early diagnosis and treatment of GI bleeding interventions is critical so that interventions can be taken before GI bleeding progresses to more severe conditions.

Recent research has shown that we must be cautious about the effects of vasoactive drug therapies on the splanchnic circulation because the effects of vasoactive agents on pHi (gastric intramucosal pH) are unpredictable (53). For example, the administration of a high dose of vasopressin may reduce rectosigmoidal mucosal perfusion and increase the risk for rectosigmoidal ischemia during and after CPB (54). Animal studies have shown that the supply of oxygen to the gastrointestinal system decreases obviously when the blood is diluted (18). Moreover, clinical outcome studies have shown an inverse relationship between mortality and the lowest hematocrit on bypass, suggesting that excessive hemodilution should be avoided (55, 56). Monitoring indicators that can be considered, such as inflammatory mediators, arterial lactic acid levels, gastrointestinal mucosal pH. In recent years, near infrared spectroscopy has been widely used in continuous monitoring of regional tissue oxygenation during perioperative and postoperative cardiopulmonary bypass in children and some pediatric studies have been conducted to determine near-infrared spectroscopy usage, safety, and efficacy (57–59). Several strategies have been proposed for improvement of CPB, such as maintaining adequate perfusion, avoiding hemodilution and severe anemia, using pulsatile blood flow, and using filters to reduce emboli. The role of pulsatile and non-pulsatile blood flow in cardiopulmonary bypass has been widely debated. Pulsating flow has improved gastrointestinal mucosal oxygenation and perfusion in some previous studies (60–62). Goal-directed therapy has been applied in a variety of perioperative and postoperative areas of medicine with promising results, usually including rigorous oxygen perfusion and monitoring and active management to improve clinical outcomes (63, 64).

The use of proton pump inhibitors to suppress gastric acid is currently recommended to reduce the risk of gastrointestinal bleeding and has been shown to be superior to non-prevention and the use of histamine receptor antagonists to prevent GI bleeding (65). The European Association of Cardiothoracic Surgery has recommended that proton pump inhibitors should be considered in all patients following heart surgery (class IIa recommendation). The improvement in prophylactic use of proton pump inhibitors was statistically significant in reducing GI bleeding after cardiac surgery (66). More cohorts are needed to further evaluate its significance.

Combined with the statistically significant correlation of these risk factors after multivariate logistic regression analysis, we can use the nomograms (Figure 2) to assess the risk of gastrointestinal bleeding after CPB in children with congenital heart disease. In our study, the area under the ROC curve of the nomograms prediction model for GI bleeding was 0.898 (95% CI: 0.884–0.914, *P* < 0.001), with a sensitivity of 80.9% and specificity of 84.2%. As a result, the nomogram has a good ability to distinguish the risk of GI bleeding after CPB in children. The risk prediction model established in this study has good sensitivity and specificity. The nomograms prediction model we developed can score patients more intuitively and quickly to predict the probability of gastrointestinal bleeding after CPB in children, which can provide certain support for clinical work.

### 4.1. Limitations

Several limitations should be considered in this study. First, this is a retrospective single-center study. Second, this study is an observational study, which can only demonstrate correlation, and further prospective studies are needed to make causal inference. Third, we noted that there may be a big difference in morbidity and mortality between asymptomatic positive fecal occult blood and a hemodynamically significant GI bleed, but the severity of GI bleeding was not measured in this observational study. The nomograms model we constructed to predict GI bleeding after CPB in children requires further external validation.

## 5. Conclusion

In this study, we identified several independent risk factors of GI bleeding post-cardiac surgery, including young age, low weight, long operation time, surgical complexity, use of ECMO, LCOS, hepatic function damage, high arterial lactate level, and low postoperative platelet counts. In particular, neonates and patients with low weight have a higher risk of developing GI bleeding. The nomograms prediction model established in this study can evaluate patients more intuitively and quickly, and predict the probability of GI bleeding after CPB operation in children, thus providing support for clinical work.

## Data Availability

The original contributions presented in the study are included in the article/**Supplementary Material**, further inquiries can be directed to the corresponding author.
