# Supplementary material for: Risk factors of gastrointestinal bleeding after cardiopulmonary bypass in children: a retrospective study
Source: Front Cardiovasc Med. 2023 Sep 19;10:1224872. doi: 10.3389/fcvm.2023.1224872 (PMC10545956; doi:10.3389/fcvm.2023.1224872)
Supplement: Supplementary file 1 [file Table1.docx]

**Table S1.** Abbreviations

| APVC | anomalous pulmonary venous connection |
| --- | --- |
| ASD | atrial septal defect |
| AVSD | atrioventricular septal defect |
| CAD | coronary artery disease |
| CoA | coarctation of aorta |
| CT | cor triatriatum |
| DORV | double outlet of right ventricle |
| HLHS | hypoplastic left heart syndrome |
| MVD | mitral valve disease |
| PA | pulmonary atresia |
| PAS | pulmonary artery sling |
| PS | pulmonary stenosis |
| PTA | persistent truncus arteriosus |
| PVS | pulmonary vein stenosis |
| SLVOT | stenosis left ventricular outflow tract |
| SRVOT | stenosis of right ventricular outflow tract |
| SV | single ventricular |
| TGA | transposition of great arteries |
| TOF | tetralogy of Fallot |
| TVD | tricuspid valve diseases |
| VSD | ventricular septal defect |
